# Using Latent Class Analysis to Identify Different Risk Patterns for Patients With Masked Hypertension

**DOI:** 10.3389/fcvm.2021.680083

**Published:** 2021-08-25

**Authors:** Ming Fu, Xiangming Hu, Shixin Yi, Shuo Sun, Ying Zhang, Yingqing Feng, Qingshan Geng, Yingling Zhou, Haojian Dong

**Affiliations:** ^1^Department of Cardiology, Guangdong Cardiovascular Institute, Guangdong Provincial People's Hospital, Guangdong Academy of Medical Sciences, The First Affiliated Hospital of South China University of Technology, Guangzhou, China; ^2^Department of Cardiology, The Second School of Clinical Medicine, Southern Medical University, Guangzhou, China

**Keywords:** masked hypertension, metabolic risk factor, community-based study, latent class analysis, risk patterns

## Abstract

**Background:** There is controversy whether masked hypertension (MHT) requires additional intervention. The aim of this study is to evaluate whether MHT accompanied with high-risk metabolic syndrome (MetS), as the subphenotype, will have a different prognosis from low-risk MetS.

**Methods:** We applied latent class analysis to identify subphenotypes of MHT, using the clinical and biological information collected from High-risk Cardiovascular Factor Screening and Chronic Disease Management Programme. We modeled the data, examined the relationship between subphenotypes and clinical outcomes, and further explored the impact of antihypertensive medication.

**Results:** We included a total of 140 patients with MHT for analysis. The latent class model showed that the two-class (high/low-risk MetS) model was most suitable for MHT classification. The high-risk MetS subphenotype was characterized by larger waist circumference, lower HDL-C, higher fasting blood glucose and triglycerides, and prevalence of diabetes. After four years of follow-up, participants in subphenotype 1 had a higher non-major adverse cardiovascular event (MACE) survival probability than those in subphenotype 2 (*P* = 0.016). There was no interaction between different subphenotypes and the use of antihypertensive medications affecting the occurrence of MACE.

**Conclusions:** We have identified two subphenotypes in MHT that have different metabolic characteristics and prognosis, which could give a clue to the importance of tracing the clinical correlation between MHT and metabolic risk factors. For patients with MHT and high-risk MetS, antihypertensive therapy may be insufficient.

## Introduction

Masked hypertension (MHT) is characterized by office readings suggesting normal BP but out-of-office readings, such as ambulatory blood pressure monitoring (ABPM) and home blood pressure monitoring, being consistently above normal (1). Meta-analysis and recent studies have shown that the risk of cardiovascular events in MHT patients is higher than in normotensive patients, whose risk is close to that of patients with persistent hypertension (2–4). With the presence of metabolic abnormalities and hypertension-mediated organ damage, the cardiovascular risk level in MHT patients might be high (5, 6). The latest arterial hypertension guideline points out that avoiding bad habits in daily life are recommended as the management for MHT to reduce cardiovascular risk (Class I, Level C), but there still lacks risk stratification of MHT to guide treatment (1).

Metabolic syndrome (MetS), as a special “disease state” resulting from an excess of stored and/or circulating energy, is associated with elevations in blood pressure (BP) (7). Although it is generally recognized that metabolic abnormalities are heterogeneous in common chronic diseases (such as diabetes and hypertension), and there is evidence that diabetic patients with different metabolic subphenotypes have different prognoses (8, 9), it is still unknown whether MHT with high-risk MetS is regarded as a subphenotype and whether it has a worse prognosis than low-risk MetS.

Latent class analysis (LCA), a well-proven statistical technique that uses mixed modeling to find the best-fit model for different populations based on baseline data classification, has been used widely to identify subphenotypes of many diseases (10). In this study, we assume that there are different subphenotypes of MHT patients, and distinguish MHT patients based on high or low-risk MetS in an LCA model to identify latent classes and observe their natural prognosis.

## Materials and Methods

This study was performed in accordance with the principles of the Helsinki Declaration II and approved by the Ethics Committee of Guangdong Provincial People's Hospital. All participants were informed and their consent to participate in this study was obtained.

### Study Population

This study was designed as a prospective cohort study consecutively enrolling MHT patients who had participated in the High-Risk Cardiovascular Factor Screening and Chronic Disease Management Programme, a project that organized by the Guangdong Provincial People's Hospital and implemented by community hospitals in Dongguan, in 2012. A follow-up registration system was established to collect data. The inclusion criteria in this study were: (1) aged 30–75; (2) diagnosed with MHT by the ABPM test; (3) left ventricular ejection fraction (Simpson's) >50%. The exclusion criteria were patients with a history of (1) secondary hypertension; (2) antihypertensive drugs used or stopped taking antihypertensive drugs for more than 2 weeks; (3) angina or myocardial infarction; (4) myocarditis; (5) stroke; (6) chronic kidney disease; and (7) autoimmune disease or tumor.

All experimental data were collected from the database of the medical center and recorded by two authors (Fu and Hu).

### Blood Pressure and Measurement

Office BP measurements in the clinic were conducted by experienced community doctors and nurses using a calibrated OMRON Upper Arm Electronic Sphygmomanometer (Model HBP1100U). After the study participants took a seat and rested for at least 5 min, the cuff blood pressure measurement obtained three consecutive readings that were averaged for analysis. ABPM was performed uniformly using the verified and qualified TM2430 Oscillometric Ambulatory Blood Pressure Monitor from A&D Company, Limited. Each participant's blood pressure was monitored for 24 h, every 20 min during the waking period and every 30 min during the sleep period. Unqualified data (including <70% success rate of blood pressure measurement or <20 readings during the waking period or <7 readings during the sleep period) were repeated.

LDL-cholesterol, HDL-cholesterol (HDL-C), triglycerides, total cholesterol and fasting blood glucose were detected using a Beckman AU5800 spectrophotometer via colorimetry. Waist circumference was measured with tape measure positioned at the level of the umbilicus when the participants stood with their feet 25–30 cm apart and at the end of the exhalation. Diabetes status was based on previous history. Smoking was defined as any previous smoking (yes/no). Alcohol consumption was defined as alcohol consumption >3 times a week (yes/no). Antihypertensive drug use was recorded according to self-report.

### Assessment of Masked Hypertension

MHT is defined as office blood pressure (average of 3 readings) <140/90 mmHg, but ambulatory blood pressure over a 24 h period with average blood pressure ≥130/80 mmHg and/or daytime (waking period) average blood pressure ≥135/85 mmHg and/or nighttime (sleeping period) average blood pressure ≥120/70 mmHg. Otherwise, patients are non-hypertensive, according to the 2013 European Society of Hypertension criteria (11).

### Long-Term Outcomes

Patients were interviewed by phone every 3 months and face-to-face every 6 months to see whether they had had any major adverse cardiovascular event (MACE), including cardiac death, stroke, or coronary heart disease (CHD). Cardiac death was defined as cardiac-caused death on the patient's death certificate. The definition of stroke was the development of neurological dysfunction symptoms and objective findings that last more than 24 h. Coronary heart disease was defined as coronary artery stenosis confirmed by coronary digital subtraction angiography equal to or more than 70%.

All patients completed a follow-up period of 48 months, and the follow-up data of patients were reviewed and evaluated in 2017. All interviews were conducted by a cardiologist, and the follow-up information was obtained from the narration of the patient or immediate relatives and further confirmed by the electronic medical record at the hospital if the patient had had a MACE.

### Statistical Analysis

In the latent class analysis model, baseline clinical data and biomarker concentrations were regarded as category-defining variables without consideration of clinical outcomes. The selection of clinical variables referred to the diagnostic criteria of metabolic syndrome, including waist circumference, HDL-C, triglycerides, fasting blood glucose, and diabetes status, in order to discriminate high/low-risk MetS. For the sake of maintaining the consistency of the direction of the variables, we converted HDL-C to 1/HDL-C, which tends to be classified as high-risk MetS when it is higher.

We first listed the clinical characteristics and laboratory data of the study population. Next, we selected waist circumference, triglycerides, HDL-C, fasting blood glucose, and diabetes status as variables to fit a series of latent class models using the depmixS4 R package (12). The model selection criterion was based on the Bayesian information criterion, log likelihood ratio test, and the size of the smallest class. The latent class model is estimated using the full-information maximum likelihood method, which allows the full use of data from all patients. Then, we identified significant differences between the groups by using the *t* test (Class = 2) or variance analysis (Class > 2) for normally distributed data, the Mann-Whitney U test (Class = 2) or Kruskal-Wallis H test (Class > 2) for skewed distribution data, and the chi-square test or Fisher's exact test for categorical variables. After that, the Kaplan-Meier method was used to describe and confirm the cumulative incidence of MACE, and the log-rank test was conducted to compare between groups. Sensitivity analysis was conducted after excluding those with diabetes status since it had a certain increased cardiovascular risk. Comparisons where *P* < 0.05 (two-sided) were considered to be statistically significant. All of the analyses were performed with Stata 15.0, R, and EmpowerStats (http://www.empowerstats.com, X&Y Solutions, Inc., Boston, MA).

## Results

### Selection of Optimal Latent Class Model

**Figure 1:**
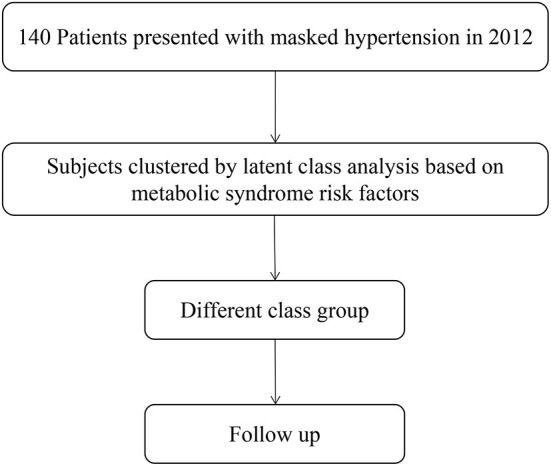
Flowchart of patient enrollment and classification.

**Figure 2:**
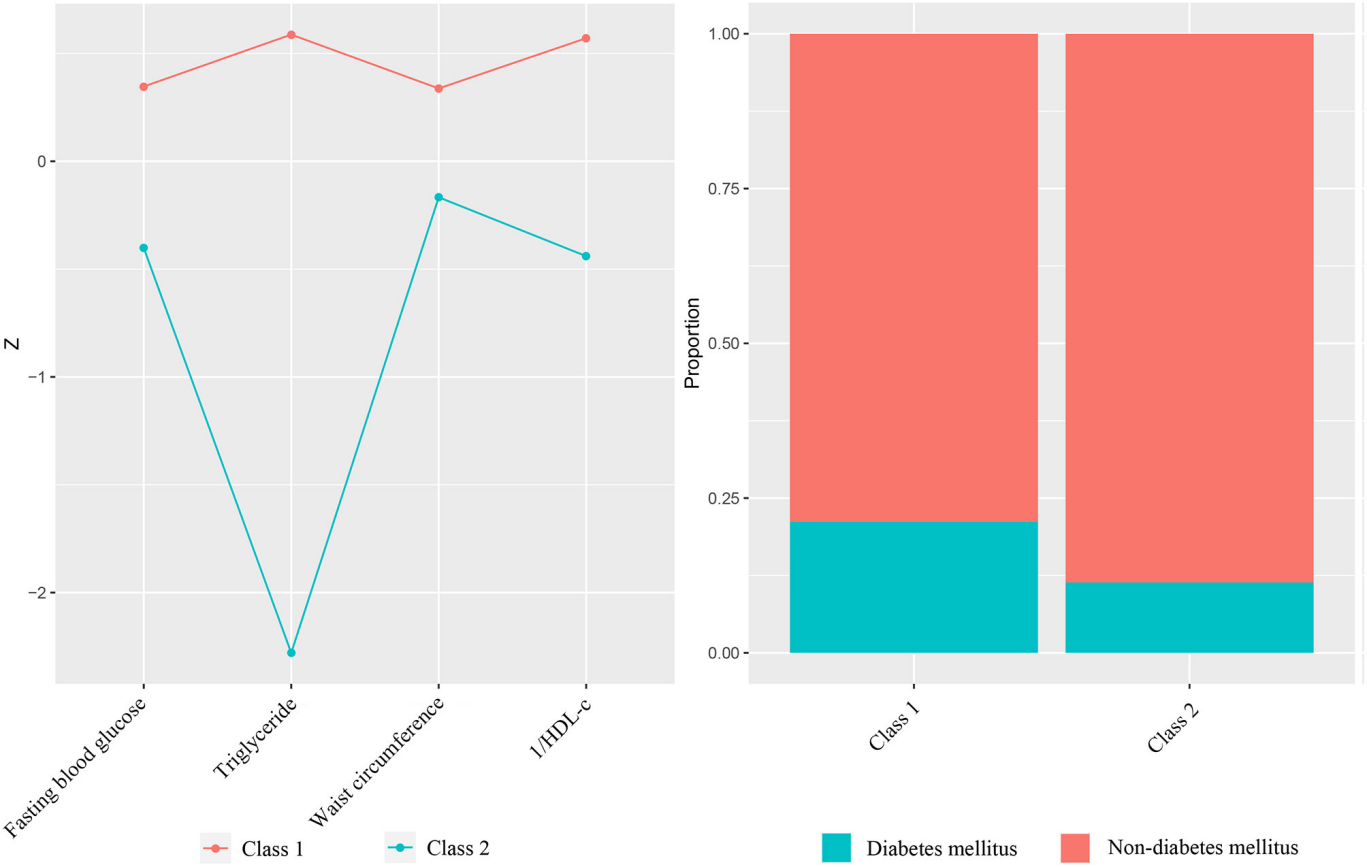
Differences of classified variables by the two-class model.

The flowchart of our study was presented in Figure 1. A total of 140 MHT patients were included in the study. Baseline clinical characteristics and laboratory tests of the patients are shown in Supplementary Table 1. The LCA showed that the *p*-value testing was significant for the two/three/four-class (Table 1). The value of Bayesian Information Criteria were lowest in three-class mode, but this model produced a class with only 28 participants. Although the reduction of the Bayesian information standard guided us to add additional classes to the model, considering our research purpose (high/low-risk classification) and the small number of participants in the three-class model, the two-class model was considered to be the best fit for the participants (Figure 2). In order to distinguish different groups conveniently, we referred to them as Class 1 and Class 2. The average latent class probabilities for the most likely class in the Class 1 were 0.93 and 0.97 for Class 2. The classification probability of 43 (82.7%) Class 1 patients and 81 (92.0%) Class 2 patients was >0.9, indicating that the model has strong grade differentiation.

**Table 1:** Summary of latent class model identification and fit statistics.

**Class**	**Bayesian information criteria**	**Number of individuals per class**				***P*-value**
		**1**	**2**	**3**	**4**	
2-class	2112.58	52	88	–	–	<0.001
3-class	294.23	28	76	36	–	<0.001
4-class	367.07	38	38	36	28	<0.001

### Population Characteristics of Different Classes

Table 2 lists the comparisons of all the variables involved in the classification. No significant difference were found in demographic spectrum and health behavior, including age, sex, smoking and alcohol consumption. Except for diabetes status, differences of classified variables were statistically significant, which again verified the reliability of the two-class model. Class 1 had a larger waist circumference, lower HDL-C, higher fasting blood glucose and triglycerides, and prevalence of diabetes, which was in line with the original intention of the high-risk MetS we set.

**Table 2:** Baseline characteristics of the study population post classification.

	**Class 1**	**Class 2**	***P*-value**
*N*	52	88	
Age	60 ± 9	58 ± 8	0.399
Male	27 (51.92%)	33 (37.50%)	0.096
Waist, cm	86.13 ± 8.28	81.68 ± 9.90	0.007
Diabetes	11 (21.15%)	10 (11.36%)	0.117
Smoking	14 (26.92%)	16 (18.18%)	0.223
Alcohol consumption	1 (1.92%)	2 (2.27%)	0.890
1/HDL-C, mg/dL	0.02 ± 0.01	0.02 ± 0.00	<0.001
Fasting blood glucose, mmol/L	5.86 ± 1.65	4.95 ± 0.84	<0.001
Triglyceride, mg/dL	263.32 ± 181.88	93.33 ± 27.70	<0.001
Waist, cm (Z score)	0.29 ± 0.87	−0.17 ± 1.04	0.007
1/HDL-C, mg/dL (Z score)	0.59 ± 1.04	−0.35 ± 0.80	<0.001
Fasting blood glucose, mg/dL (Z score)	0.45 ± 1.29	−0.26 ± 0.66	<0.001
Triglyceride, mg/dL (Z score)	0.77 ± 1.31	−0.45 ± 0.20	<0.001

*Values are shown as mean ± SD or n (%). HDL-C, high-density lipoprotein cholesterol*.

### Prognoses of Different Classes

**Figure 3:**
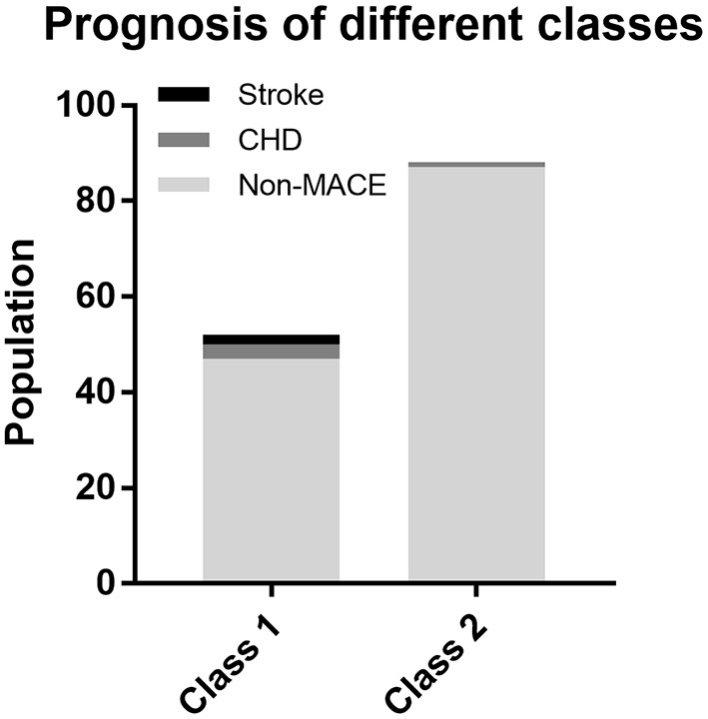
Composition of outcome at 48 months follow-up by different classes.

**Figure 4:**
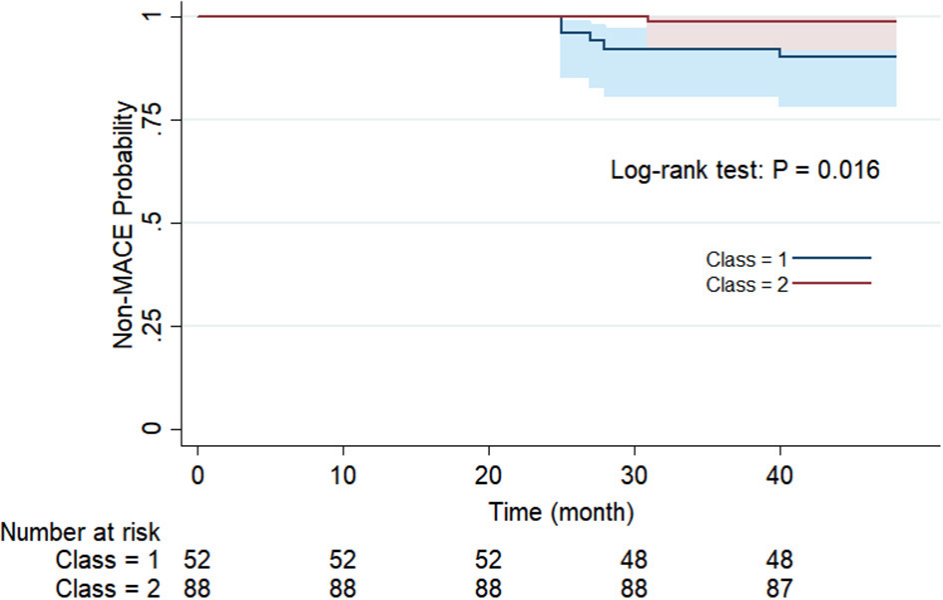
Survival analysis of different classes at 48 months follow-up.

For the purpose of determining whether these two categories have different natural prognoses, we did the survival analysis based on the 48-month follow-up information. Figure 3 illustrates the composition of the outcome, where a total of five people in Class 1 had MACE, of which two had stroke and three had CHD, while one case in Class 2 had CHD. There was no occurrence of cardiac death in either class. As shown in Figure 4, overall MACE-free survival at 48 months was 90.4% in Class 1 and 98.9% in Class 2 (Log-rank test: *P* = 0.016). Sensitivity analysis also suggested that after excluding those patients with diabetic status (*n* = 21), the natural prognosis of difference classes still significant (Log-rank test: *P* = 0.009, Supplementary Figure 1). Furthermore, in order to clarify whether inter-class differences existed in the prognosis after blood pressure-lowering medications, we tested the interaction. The result suggested that there was no statistically significant interaction between different subphenotypes and blood pressure-lowering drug use (Supplementary Table 2).

## Discussion

Our findings suggested that in MHT patients, there are two different classes that have various clinical features, pathophysiological characteristics, and natural prognoses, meeting the necessary criteria for defining subphenotypes. Although we have recognized the heterogeneity of many chronic diseases in the past, there is currently no clinical cohort study for MHT to clarify its potential classification and further guide effective countermeasures. In our study, there was a significant correlation between subphenotypes and clinical results, which indicated that the identification of these two subphenotypes may be crucial for future MHT clinical interventions.

The demographic characteristics of the classes reflected the uniformity of allocation, while the MetS-associated classified variables discriminated between the high- and low-risk MetS subphenotypes. Overall, phenotype 1, as an identification group, described the patients with larger waist circumference, less HDL-C, higher fasting blood glucose, triglycerides, and prevalence of diabetes. In contrast, phenotype 2 showed characteristics opposite to phenotype 1 and belonged to low-risk MetS patients. What is interesting is that the natural classification of all participants was in line with the diagnostic criteria of MetS, which was a reasonable clustering method and corresponded with clinical practice. In LCA, the best-fit model was based on a given number of categories. Although the *P* value of the log likelihood ratio test was significant among the two/three/four-class in our research, considering the population distribution, posterior probability of allocation, and especially the original intention of our research design, the two-class model was finally adopted.

Clustering of cardiac metabolic risk factors in adults is known as “metabolic syndrome,” a concept that is useful in both clinical and research fields. The mechanisms of the relationship between metabolic risk factors and elevated blood pressure are still unclear. A cross-sectional study conducted by Afsar indicated that the prevalence of MetS was increased in patients with MHT more than in normotensive individuals, and it was highest in patients with persistent hypertension (13). The Ohasama Study conducted a population-based survey of 395 people and reported that elevated blood pressure was significantly associated with clustering of metabolic risk factors (14). Furthermore, a systematic review suggested that patients with MetS have a higher risk of non-dipping blood pressure than patients without MetS (15). These discoveries increased the possibility that metabolic risk factors may contribute to the occurrence of hypertension. In terms of specific metabolic factors, Mancia et al. confirmed that serum glucose has been associated with the development of hypertension, and also proposed that MHT associated with metabolic risk factors is likely to worsen hypertension, thereby contributing to a poor prognosis (16). Kenny et al. emphasized the importance of waist circumference and proposed that patients with MHT were more likely to have metabolic syndrome and high-normal office blood pressure compared with normotensive people (17). Another study also showed that diabetes status and insulin levels are related to elevated blood pressure (18). Although the pathogenesis of the pathophysiological relationship between metabolic risk factors and elevated blood pressure has not been well clarified, we already know that being overweight will not only lead to abnormal structure and function of many organs and systems in the body, but will also increase all-cause mortality, including cardiovascular disease (19, 20). A tendency to obesity or existing abdominal obesity can activate the renin-angiotensin-aldosterone system, causing expression of inflammatory factors and free radicals and increased arterial resistance, which may be the cause of secondary dyslipidemia, elevated fasting plasma glucose, insulin resistance, and high blood pressure (18). In recent years, epicardial adipose tissue has received widespread attention. It is also related to hypertension, which also illustrates the inherent correlation between MetS-related lipid metabolism abnormalities and the occurrence of atherosclerotic hypertension (21). In summary, most studies support the suggestion that elevated blood pressure is a secondary change of metabolic disorders. That is, some insidious cases of hypertension may be derived from early metabolic disorders. Therefore, our research divided MHT into high/low-risk MetS categories, to distinguish those cases of MHT driven by metabolic disorders. Our results agreed with the conclusions of previous studies.

In addition to the methodological advantages of latent category analysis, another strength of our study was the four-year follow-up of the two classes of patients. The follow-up information was provided by dozens of community hospitals where every enrolled patient had completed their follow-up interviews. The results showed that more MACE occurred in Class 1; in particular, there were increased incidences of stroke and CHD. A large cohort study which recruited more than 2 million people found an obvious dose-response relationship between cumulative exposure to metabolic disorders and stroke, as people with three metabolic factors had a 24% increased risk of stroke (22). As for CHD, a Mendelian randomization study suggested that the loci associated with the direct action of HDL-C and triglycerides appeared to have location- and mechanism-specific causality on coronary artery disease (23). All of this evidence from the genetic level and high-quality population studies help explain why the incidence of coronary heart disease and stroke in Class 1 (high-risk MetS) is higher than in Class 2 (24).

The impact of subphenotype on medication choice is also of concern, so we conducted an interaction analysis, using data on the use of antihypertensive drugs. The results indicated that there was no significant difference in subphenotype between antihypertensive treatment and the occurrence of MACE, which may be due to the reason that antihypertensive drugs did not address the problem of metabolic disorders. As a kind of antihypertensive drugs, mineralocorticoid receptor antagonists are considered to be clinically valuable treatments for metabolic disorders in hypertensive patients. This is because obesity, as the center of MetS, stimulates the secretion of mineralocorticoid which is associated with an increased risk of insulin resistance, metabolic syndrome, and diabetes (25). Mineralocorticoid receptor antagonists can not only lower blood pressure, but also improve insulin sensitivity, relieve oxidative stress and reduce the inflammatory response of MetS, which could eventually prevent the occurrence of cardiovascular disease (26). In our study, however, since there was a small percentage of patient who took mineralocorticoid receptor antagonists, the protective effect on cardiovascular disease may not be well manifested. From this point of view, on the basis of lowering blood pressure, the management of metabolic disorders are needed to be considered in MHT patient with high-risk MetS.

This study has some limitations. First, our research was performed on a small sample within a single city; thus, generalization of the conclusions must be limited. Second, the boundary settings of the categorical variables were based on the characteristics of the population; that is, no specific cut off values were defined for the boundaries, which may render the variables impractical as clinical tools. However, what is worth mentioning is that compared with subphenotype discrimination of other diseases, the number of categorical variables used in this study was relatively few. Third, although these metabolic risk factors are valuable for prognosis, rapid developments in metabolic genetics and novel molecular markers may facilitate more comprehensive identification of subphenotypes.

## Conclusion

Classification based on metabolic risk factors suggests that MHT contains different subphenotypes and has different prognoses, prompting a need for future large-scale cohort studies to further elucidate these subphenotypes. In addition to antihypertensive treatment, the management of metabolic disorder in MHT populations may be also considered in the future.

## Data Availability Statement

The raw data supporting the conclusions of this article will be made available by the authors, without undue reservation.

## Ethics Statement

The studies involving human participants were reviewed and approved by Ethics Committee of Guangdong Provincial People's Hospital. The patients/participants provided their written informed consent to participate in this study.

## Author Contributions

MF and XH prepared and wrote the manuscript. SY and SS performed the data analysis. YZha and YF contributed to the acquisition of the data. QG reviewed and made critical revisions to the manuscript. YZho and HD contributed to the ideas and approved the final version of the manuscript. All authors have read and approved the final manuscript.

## Conflict of Interest

The authors declare that the research was conducted in the absence of any commercial or financial relationships that could be construed as a potential conflict of interest.

## Publisher's Note

All claims expressed in this article are solely those of the authors and do not necessarily represent those of their affiliated organizations, or those of the publisher, the editors and the reviewers. Any product that may be evaluated in this article, or claim that may be made by its manufacturer, is not guaranteed or endorsed by the publisher.

## References

[1] Williams B Mancia G Spiering W Agabiti Rosei E Azizi M Burnier M. 2018 ESC/ESH Guidelines for the management of arterial hypertension. Eur Heart J. (2018) 39:3021–104. 10.1093/eurheartj/ehy339 30165516

[2] Zhang DY Guo QH An DW Li Y Wang JG. A comparative meta-analysis of prospective observational studies on masked hypertension and masked uncontrolled hypertension defined by ambulatory and home blood pressure. J Hypertens. (2019) 37:1775–85. 10.1097/HJH.0000000000002109 31219948

[3] Mancia G Facchetti R Bombelli M Grassi G Sega R. Long-term risk of mortality associated with selective and combined elevation in office, home, and ambulatory blood pressure. Hypertension. (2006) 47:846–53. 10.1161/01.HYP.0000215363.69793.bb 16567588

[4] Fagard RH Cornelissen VA. Incidence of cardiovascular events in white-coat, masked and sustained hypertension versus true normotension: a meta-analysis. J Hypertens. (2007) 25:2193–8. 10.1097/HJH.0b013e3282ef6185 17921809

[5] Tientcheu D Ayers C Das SR McGuire DK de Lemos JA Khera A. Target organ complications and cardiovascular events associated with masked hypertension and white-coat hypertension: analysis from the Dallas heart study. J Am Coll Cardiol. (2015) 66:2159–69. 10.1016/j.jacc.2015.09.007 26564592 PMC4644495

[6] Mancia G Bombelli M Facchetti R Madotto F Quarti-Trevano F Grassi G. Increased long-term risk of new-onset diabetes mellitus in white-coat and masked hypertension. J Hypertens. (2009) 27:1672–8. 10.1097/HJH.0b013e32832be5f9 19417688

[7] Ecelbarger CM. Metabolic syndrome, hypertension, and the frontier between. Am J Physiol Renal Physiol. (2016) 310:F1175–7. 10.1152/ajprenal.00095.2016 26911845

[8] DeBoer MD Filipp SL Gurka MJ. Use of a metabolic syndrome severity Z score to track risk during treatment of prediabetes: an analysis of the diabetes prevention program. Diabetes Care. (2018) 41:2421–30. 10.2337/dc18-1079 30275282 PMC6196828

[9] Chuang SM Shih HM Chien MN Liu SC Wang CH Lee CC. Risk factors in metabolic syndrome predict the progression of diabetic nephropathy in patients with type 2 diabetes. Diabetes Res Clin Pract. (2019) 153:6–13. 10.1016/j.diabres.2019.04.022 31063854

[10] Kongsted A Nielsen AM. Latent class analysis in health research. J Physiother. (2017) 63:55–8. 10.1016/j.jphys.2016.05.018 27914733

[11] O'Brien E Parati G Stergiou G Asmar R Beilin L Bilo G. European Society of Hypertension position paper on ambulatory blood pressure monitoring. J Hypertens. (2013) 31:1731–68. 10.1097/HJH.0b013e328363e964 24029863

[12] Visser I Speekenbrink M. depmixS4: An R package for hidden Markov models. J Stat Softw. (2010) 36:1–21. 10.18637/jss.v036.i07

[13] Afsar B. Comparison of demographic, clinical, and laboratory parameters between patients with sustained normotension, white coat hypertension, masked hypertension, and sustained hypertension. J Cardiol. (2013) 61:222–6. 10.1016/j.jjcc.2012.11.003 23294898

[14] Sato A Asayama K Ohkubo T Kikuya M Obara T Metoki H. Optimal cutoff point of waist circumference and use of home blood pressure as a definition of metabolic syndrome: the Ohasama study. Am J Hypertens. (2008) 21:514–20. 10.1038/ajh.2007.88 18437142

[15] Pierdomenico SD Cuccurullo F. Ambulatory blood pressure monitoring in type 2 diabetes and metabolic syndrome: a review. Blood Press Monit. (2010) 15:1–7. 10.1097/MBP.0b013e3283360ed1 20071977

[16] Mancia G Bombelli M Facchetti R Madotto F Quarti-Trevano F Polo Friz H. Long-term risk of sustained hypertension in white-coat or masked hypertension. Hypertension. (2009) 54:226–32. 10.1161/HYPERTENSIONAHA.109.129882 19564548

[17] Kenny IE Saeed S Gerdts E Midtbø H Halland H Lønnebakken MT. Masked hypertension in obesity: potential predictors and arterial damage. Blood Press Monit. (2017) 22:12–7. 10.1097/MBP.0000000000000220 27776078 PMC5213843

[18] Björklund K Lind L Zethelius B Andrén B Lithell H. Isolated ambulatory hypertension predicts cardiovascular morbidity in elderly men. Circulation. (2003) 107:1297–302. 10.1161/01.CIR.0000054622.45012.12 12628951

[19] Flegal KM Kit BK Orpana H Graubard BI. Association of all-cause mortality with overweight and obesity using standard body mass index categories: a systematic review and meta-analysis. JAMA. (2013) 309:71–82. 10.1001/jama.2012.113905 23280227 PMC4855514

[20] Bhaskaran K Dos-Santos-Silva I Leon DA Douglas IJ Smeeth L. Association of BMI with overall and cause-specific mortality: a population-based cohort study of 3.6 million adults in the UK. Lancet Diabetes Endocrinol. (2018) 6:944–53. 10.1016/S2213-8587(18)30288-2 30389323 PMC6249991

[21] Sengul C Cevik C Ozveren O Duman D Eroglu E Oduncu V. Epicardial fat thickness is associated with non-dipper blood pressure pattern in patients with essential hypertension. Clin Exp Hypertens. (2012) 34:165–70. 10.3109/10641963.2011.577488 22008026

[22] Lee EY Han K Kim DH Park YM Kwon HS Yoon KH. Exposure-weighted scoring for metabolic syndrome and the risk of myocardial infarction and stroke: a nationwide population-based study. Cardiovasc Diabetol. (2020) 19:153. 10.1186/s12933-020-01129-x 32993664 PMC7525999

[23] Thomas DG Wei Y Tall AR. Lipid and metabolic syndrome traits in coronary artery disease: a Mendelian randomization study. J Lipid Res. (2020) 62:100044. 10.1194/jlr.P120001000 32907989 PMC7933489

[24] Konishi M Sugiyama S Sugamura K Nozaki T Matsubara J Akiyama E. Accumulation of pericardial fat correlates with left ventricular diastolic dysfunction in patients with normal ejection fraction. J Cardiol. (2012) 59:344–51. 10.1016/j.jjcc.2012.01.006 22365950

[25] Whaley-Connell A Johnson MS Sowers JR. Aldosterone: role in the cardiometabolic syndrome and resistant hypertension. Prog Cardiovasc Dis. (2010) 52:401–9. 10.1016/j.pcad.2009.12.004 20226958 PMC2841057

[26] Tirosh A Garg R Adler GK. Mineralocorticoid receptor antagonists and the metabolic syndrome. Curr Hypertens Rep. (2010) 12:252–7. 10.1007/s11906-010-0126-2 20563672 PMC2948675

