# Supplementary material for: Using Latent Class Analysis to Identify Different Risk Patterns for Patients With Masked Hypertension
Source: Front Cardiovasc Med. 2021 Aug 25;8:680083. doi: 10.3389/fcvm.2021.680083 (PMC8424076; doi:10.3389/fcvm.2021.680083)

Supplementary Table 1. Baseline characteristics of the study population at enrollment.

| N | 140 |
| --- | --- |
| Age, year | 58.79 ± 8.41 |
| Body Mass Index | 24.37 ± 3.36 |
| Male | 60 (42.86%) |
| Waist, cm | 83.33 ± 9.55 |
| Fasting blood glucose, mmol/L | 5.29 ± 1.28 |
| [T](../../../../E:/%25E8%25BD%25AF%25E4%25BB%25B6/%25E6%259C%2589%25E9%2581%2593/Youdao/Dict/8.9.6.0/resultui/html/index.html#/javascript:;)otal [cholesterol](../../../../E:/%25E8%25BD%25AF%25E4%25BB%25B6/%25E6%259C%2589%25E9%2581%2593/Youdao/Dict/8.9.6.0/resultui/html/index.html#/javascript:;), mg/dL | 203.12 ± 36.82 |
| [T](../../../../E:/%25E8%25BD%25AF%25E4%25BB%25B6/%25E6%259C%2589%25E9%2581%2593/Youdao/Dict/8.9.6.0/resultui/html/index.html#/javascript:;)riglyceride, mg/dL | 156.47 ± 139.33 |
| LDL-C, mg/dL | 97.32 ± 23.43 |
| HDL-C, mg/dL | 49.63 ± 13.28 |
| Uric acid, μmol/l | 407.44 ± 121.76 |
| Diabetes | 21 (15.00%) |
| Smoking | 30 (21.43%) |
| Alcohol | 3 (2.14%) |
| Antihypertensive drug |  |
| ACEI | 15 (10.7%) |
| ARB | 69 (49.2%) |
| CCB | 63 (45.0%) |
| Diuretic | 6 (4.2%) |
| β- blocker | 10 (7.1%) |

Notes: Values are shown as mean ± SD or n (%). Abbreviation: LDL-C: low-density lipoprotein cholesterol; HDL-C: high-density lipoprotein cholesterol. ACEI: angiotensin-converting enzyme inhibitors; ARB: angiotensin Receptor Blockers; CCB: calcium channel blockers.

Supplementary Table 2. Effect of interaction between different subphenotypes and antihypertensive medication on the occurrence of MACE.

| Class | Antihypertensive drug | | P for interaction |
| --- | --- | --- | --- |
|  | Yes | No |  |
| 1 | 46 | 6 | 0.3114 |
| 2 | 61 | 27 |  |

Supplementary Figure 1. Survival analysis of different classes at 48 months follow-up after excluding those with diabetes status.


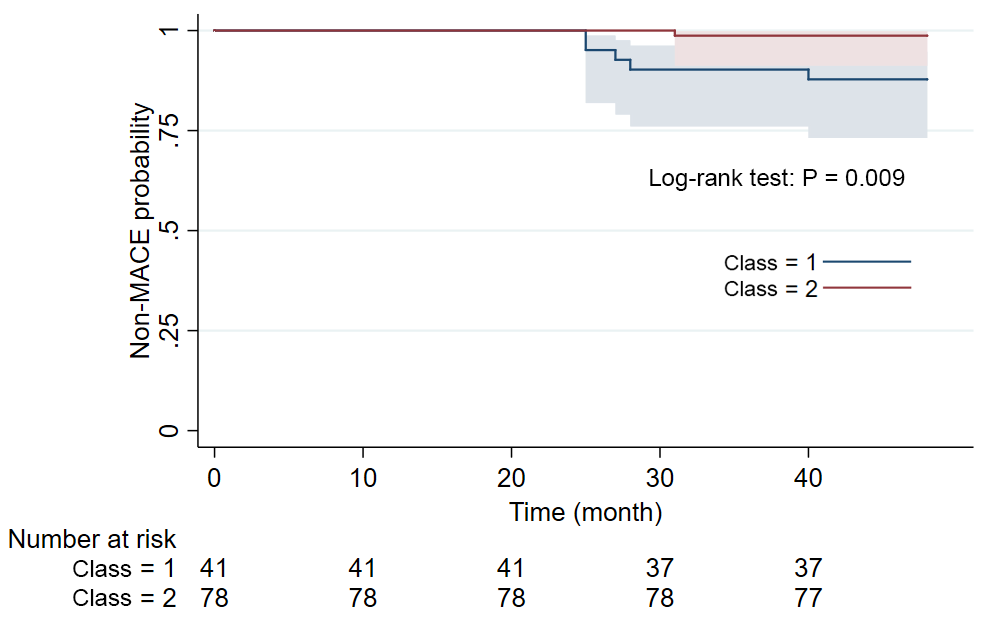

Supplement: Supplementary file 1 [file Data_Sheet_1.doc]
